# Let’s do it: Response times in Mental Paper Folding and its execution

**DOI:** 10.1177/17470218241249727

**Published:** 2024-05-12

**Authors:** Stephan Frederic Dahm, Pierre Sachse

**Affiliations:** Department of Psychology, Faculty of Psychology and Sports Sciences, University of Innsbruck, Innsbruck, Austria

**Keywords:** Movement execution, motor imagery, action imagery, mental chronometry

## Abstract

Action imagery is the ability to mentally simulate the execution of an action without physically performing it. Action imagery is assumed to rely at least partly on similar mechanisms as action execution. Therefore, we expected that imagery and execution durations would be constrained by the number of folds in a Paper Folding Task. Analogously, individual differences in execution durations were expected to be reflected in imagery durations. Twenty-eight participants performed two imagery conditions (computer vs. paper) and one execution condition (paper) where two-dimensional grids of a three-dimensional cube were (mentally) folded to determine whether two selected edges overlapped or not. As expected, imagery performance and execution performance were strongly correlated and decreased with the number of folds. Further, the number of folds influenced imagery durations even more than execution durations. This may be due to the additional cognitive load in imagery that emerges when tracking the folds to follow up with the next ones. The results indicate that Mental Paper Folding predominantly involves dynamic visual representations that are not functionally associated with one’s own movements as in action imagery.

## Introduction

Action imagery (also called motor imagery) refers to a mental simulation of an action and its consequences (Dahm & Rieger, 2019; Kilteni et al., 2018), without actually performing it (Jeannerod, 1995). Some approaches assume functional similarity (Jeannerod, 2001) and similar mechanisms (Rieger et al., 2023) between action execution and action imagery while others emphasise the differences (Glover & Baran, 2017; Guillot, Di Rienzo, et al., 2012; Rieger et al., 2017). Using the mental chronometry paradigm, the present study compares action imagery durations and action execution durations in a Paper Folding Task (Dahm & Draxler, 2022). Further, Mental Paper Folding Performance is related to a prevailing self-judgement questionnaire of action imagery (Dahm, 2022; Roberts et al., 2008). In addition, potential underlying processes of Mental Paper Folding such as intelligence and task-specific self-ratings are examined.

Action imagery differs from (visual) imagery (e.g. “imagine a black table”) in a way that it involves the visualisation of effort, encompassing various modalities associated with one’s own action such as kinesthetic, tactile, visual, and acoustic elements (Cumming & Eaves, 2018; Dahm, 2020; Krüger et al., 2022; Lacey & Lawson, 2013). Instead of the more common term *motor imagery*, we use the term *action imagery* to emphasise that imagining movements goes beyond kinesthetic (motor) representations involving visual aspects at the same time. Visual action imagery can manifest either from a first-person perspective (“through the own eyes”) or a third-person perspective (“from another viewpoint”). In addition, action imagery encompasses considerations of the consequences of the action (Dahm & Rieger, 2019; Kilteni et al., 2018; Rieger et al., 2023) which are essential in goal-directed action execution (Elsner & Hommel, 2001; Hommel, 2009; Hommel et al., 2001). It is important to note that individuals may vary in their ability to imagine actions and may exhibit distinct preferences regarding perspectives and modalities. From an applied point of view, this is of particular relevance, as individuals with high action imagery ability benefit more from subsequent action imagery practice than individuals with low action imagery ability (Yoxon et al., 2022).

### Similarities in action imagery and action execution

To gain a better understanding of the common and unique mechanisms of action execution and action imagery and to describe them more precisely, it is essential to understand the mechanisms involved in *action execution*. When an action is intended, certain consequences are intended (Bach et al., 2022). Based on the intended action consequences and the given affordance, inverse models select the appropriate motor commands (Rieger et al., 2023). Subsequently, effectors are activated, and efference copies of the motor commands are created. Using these efference copies, *forward models* can predict the consequences. If there are discrepancies between the predicted and intended action consequences, inverse models adjust the corresponding motor commands (Blakemore et al., 2002; Davidson & Wolpert, 2005). In action execution, forward modelling makes it possible to detect errors even before they occur to adjust the action (Maidhof et al., 2009). In addition, changes to the body and environment occur as a result of the upcoming action. The acting person observes these consequences using sensory feedback and compares them to the intended and predicted consequences.

The execution of an action includes two phases (Heuer et al., 1998). During *movement preparation*, the action is planned and the movement parameters are determined. During *movement execution*, the muscles are activated and the action becomes externally observable. The preparation phase is considered an unconscious way of imagining the action (Jeannerod, 1995). Jeannerod’s (1995) Simulation Theory considers action imagery as representations of actions associated with movement preparation and planning processes. It is considered a subliminal activation of the motor system and causes inverse models to select motor commands according to the intended goal of the action (Rieger et al., 2023). This implies that even without actual feedback, representations of the action and its consequences are encoded to detect deviations from the intended consequences (Dahm & Rieger, 2019).

*Functional brain imaging* studies support Jeannerod’s (1995) assumption that action imagery and action execution are functionally similar. For instance, it has been shown that action imagery activates the corresponding body-part-specific motor areas in the brain (Ehrsson et al., 2003). Although brain areas active during action imagery and action execution overlap (Hanakawa et al., 2003; Lorey et al., 2013; Macuga et al., 2012), this is not a complete overlap (Hardwick et al., 2017; Hétu et al., 2013). In fact, there are areas that are active during the execution of an action but not during its imagination and vice versa (Dietrich, 2008). The differences possibly arise from the fact that during action imagery, actual execution must be inhibited (Dietrich, 2008; Guillot, Di Rienzo, et al., 2012; Rieger et al., 2017), which prevents the execution of motor commands (Rieger et al., 2023).

*Behavioural studies* provide further evidence for functional similarity conducted using the *Mental Chronometry Paradigm* (Dahm & Rieger, 2016a, 2016b; Guillot & Collet, 2005; Guillot, Hoyek, et al., 2012). Here, the durations of executing and imagining the same action are compared. The durations are assumed to reflect the progress in planning and executing the action while similar durations shall be caused by similar underlying neural mechanisms. However, durations oftentimes deviate (Guillot, Hoyek, et al., 2012) because action imagery requires a focus on processes that are automated and unconscious in action execution (Glover & Baran, 2017). By this, the Motor-Cognitive Model postulates executive functions in action imagery that are not required in action execution (Glover et al., 2020). Indeed, it has been shown that disrupting executive function via Transcranial Magnetic Stimulation increases durations in action imagery, but not in action execution (Martel & Glover, 2023). Although the absolute durations overlap only infrequently (Decety, 1991; Grealy & Shearer, 2008), constraining factors that influence execution durations have often been shown to influence action imagery durations similarly (Guillot & Collet, 2005; Guillot, Hoyek, et al., 2012). For instance, comparable bimanual coordination constraints were observed on action imagery and execution durations, but action imagery took longer than action execution (Dahm & Rieger, 2016b). Similar results were found for cognitive constraints, where action imagery and action execution were comparably influenced, but action imagery lasted longer than action execution (Dahm & Rieger, 2016a). Other constraints may include, for example, differences in right and left-hand writing speed (Decety & Michel, 1989), movement difficulties (Decety & Jeannerod, 1995), physical laws such as inertia and gravity (Papaxanthis et al., 2002, 2003) and tool characteristics such as the thickness of a pen (Rieger & Massen, 2014). Furthermore, it was shown that the durations of imagined and executed actions correlate with each other (Dahm & Rieger, 2016a; Decety et al., 1989; Rieger, 2012; Williams et al., 2015). Hence, individual differences in execution time are also reflected in action imagery.

### Relation between action imagery and intelligence

A highly related construct in the nomological net to action imagery is *intelligence* (Kunda, 2020), which involves the ability to quickly perceive and process information to give a response (Meiran & Shahar, 2018). Logical matrix reasoning is often used as a nonverbal estimate of fluid intelligence, which is a primary factor of spatial imagery and abstract spatial reasoning (Heydasch et al., 2013). Hence, spatial reasoning is a cognitive ability similar to fluid reasoning and therefore a component of intelligence that includes mental rotation, spatial visualisation, and matrix thinking (King et al., 2019; Linn & Petersen, 1985; Shepard & Metzler, 1971). By this, spatial reasoning enables us to make inferences about 2D and 3D objects (King et al., 2019). The more complex the task, the more strongly spatial visualisation tends to correlate with fluid intelligence. For instance, the Ekstrom paper folding test, which is a complex spatial visualisation task (Miyake et al., 2001) is associated to fluid intelligence (King et al., 2019).

### Mental Paper Folding Task

In the Mental Paper Folding Task (Shepard & Feng, 1972), a two-dimensional grid of squares is shown, which can be folded into a three-dimensional cube. Participants are asked to indicate whether two highlighted lines in the two-dimensional grid would overlap if they were folded into a three-dimensional cube (Dahm & Draxler, 2022). Mental Paper Folding may be considered an appropriate objective method to assess action imagery ability because it involves all four dimensions of action imagery (Cumming & Eaves, 2018; Kosslyn et al., 1984). The first dimension, *Image Creation*, involves the creation of an imagined perception (visual, auditory, etc.) using stored representations. In Mental Paper Folding, image creation is supported by the visual input of the two-dimensional grid. The second dimension, *Image Inspection*, deals with focusing on details for information interpretation and extraction. In the folding task, it plays a crucial role in the final decision whether there is an overlap of the highlighted lines or not. The third dimension, *Image Transformation*, involves the intentional manipulation of the imagined content and describes the continuous process of the action itself. The imagined folding of a cube is an apt example of this dimension. The fourth dimension, *Image Maintenance* or *Controllability*, refers to the ability to preserve the imagined perception. This is especially important for grids that require a high number of folds, to consistently retain the image of the last transformation. These four dimensions of action imagery ability correspond to the concept of Mental Paper Folding, as each of these dimensions is needed to visualise and perform the entire action.

In the Mental Paper Folding Task, response accuracy decreases as more relevant folds are required (Dahm & Draxler, 2022; Shepard & Feng, 1972). Further, empirical evidence suggests that regardless of response accuracy, response times (RTs) provide additional information about the individual’s underlying strategies or abilities in solving the task (Draxler & Dahm, 2020). For instance, RTs have been shown to increase linearly with the number of relevant folds that would have been required if the action had actually been performed (Dahm & Draxler, 2022; Shepard & Feng, 1972). Therefore, action imagery may be considered to occur spontaneously when it is implicitly required despite the absence of explicit prompting (Burte et al., 2019; Dahm & Draxler, 2022; Harris et al., 2013). The fact that RTs increase with distance between target squares (Shepard & Feng, 1972) is consistent with data showing that imagined walking time increases with increasing walking distance (Decety et al., 1989) and that imagined Movement Time increases with an increasing number of key presses (Dahm & Rieger, 2016b). However, the assumed actual increase has not been tested so far in the Mental Paper Folding Task. Therefore, participants actually executed the Paper Folding Task in the present study. The standard Mental Paper Folding Task is best to be assessed on the computer to allow for reliable response time measures. However, to control for possible differences between paper folding on the computer (Virtual Imagery) and paper folding with real paper (Execution), we added a second imagery version (Realistic Imagery with real paper). Differences between the imagery versions could arise from starting the timer which occurs automatically in the virtual version when the stimulus is presented but needs to be done by the participants in the realistic version. Further, the realistic stimulus material might facilitate the task compared to the more abstract stimuli on the computer (McMillan et al., 2023; Sachse & Hacker, 2012).

There are several causes that can lead to errors in the Mental Paper Folding Task. Low action imagery ability (or a lack of mental rotation skills) may result in increased guessing tendencies or losing controllability of the mental representation during action imagery. Alternatively, the representation may have been detailed and stable, but the action itself may have been imagined incorrectly, just as action errors occur during execution (Dahm & Rieger, 2019). By all means, higher error rates (ERs) are expected in action imagery than in action execution because image maintenance is not required during execution as the object is visible at all stages. Moreover, the higher load in imagery may increase RTs compared to execution performance (Glover et al., 2020). We therefore expected longer RTs in imagined paper folding than in executed paper folding. Further, we expected correlations between action-specific (the Paper Folding Task) and action-unspecific imagery ability ratings (Dahm, 2022; Roberts et al., 2008) and imagined paper folding durations, but not with executed paper folding durations.

In addition, Mental Paper Folding has been shown to activate brain areas associated with higher cognitive functions (Gevins, Zeitlin, Doyle, et al., 1979; Gevins, Zeitlin, Yingling, et al., 1979) and the processing of spatial information about orientation and positioning (Andersen et al., 1997; Glass et al., 2013), as well as perception and object recognition (Schendan & Stern, 2007). Hence, we expected a correlation of Mental Paper Folding Performance (Dahm & Draxler, 2022) with visual-spatial intelligence (Heydasch et al., 2013).

## Methods

### Participants

The required sample size was estimated with G*Power (Faul et al., 2007) for a repeated measures ANOVA with three action conditions (Virtual Imagery, Realistic Imagery, Execution). We assumed a medium effect size of η_*p*_² = .25 and a correlation between conditions of *r* = .5. Alpha was set at .05 and the power (1-beta) at .8 which resulted in a minimum sample size of *N* = 28.

Of 34 collected data sets, the data of two participants were not taken into analysis due to a test score of eight on the infrequency scale (Maniaci & Rogge, 2014) which indicates inattentive responses (e.g. reporting low values on “It feels good to be appreciated”). Further, four participants were not taken into analysis due to very high RTs (> 3 *SD*) in the imagery conditions. The remaining 28 participants (17 female) were between 19 and 28 years old (*M* *±* *SD* = 23.3 ± 1.8 years). The Laterality Index assessed with the Edinburgh Handedness Inventory (Oldfield, 1971) ranged from −100 to + 100 (*M* *±* *SD* = 68 ± 61), indicating mainly right-handers. The German version (Dahm, 2022) of the Vividness of Movement Imagery Questionnaire (Roberts et al., 2008) indicated high action imagery abilities in External Visual Imagery (*M* *±* *SD* = 2.3 ± 0.9), Internal Visual Imagery (*M* *±* *SD* = 2.1 ± 0.9), and Kinesthetic Imagery (*M* *±* *SD* = 2 ± 0.8). All participants gave informed consent and received course credit for their participation. The study was conducted in accordance with the Belmont Report (The Belmont Report, 1978).

### Item selection for the Mental Paper Folding Task

To increase item discrimination, the most difficult items were selected by using data from previous experiments (Dahm & Draxler, 2022). Two items were selected for each possible combination of Folds (Three folds, Four folds, and Five folds), Direction Changes (Fewer and More), and Overlaps (Overlap and No overlap). This resulted in 24 items. Because a random order of the items would not have been feasible in the realistic versions of the task, a fixed order was created. Based on face validity of the experimenter, the items were ordered in a way that consecutive items differed in the appearance of the base grid, Direction Changes, Overlaps, and Folds.

### Procedure

The experiment was run using OpenSesame version 3.3.14 (Mathôt et al., 2012). The experimental file including all stimuli and instructions is placed at the Open Science Framework (https://osf.io/fk7ae). Participation lasted approximately 45 min.

For familiarisation with the response arrow keys, participants performed a Choice Reaction Time Task (three choice decision task) where they were asked to respond to visual stimuli (left arrow, down arrow, and right arrow; size of 1 cm in the centre of the screen) by pressing the corresponding key on the keyboard. The stimuli were presented in random order with each stimulus being presented four times (12 trials in total).

**Figure 1.**
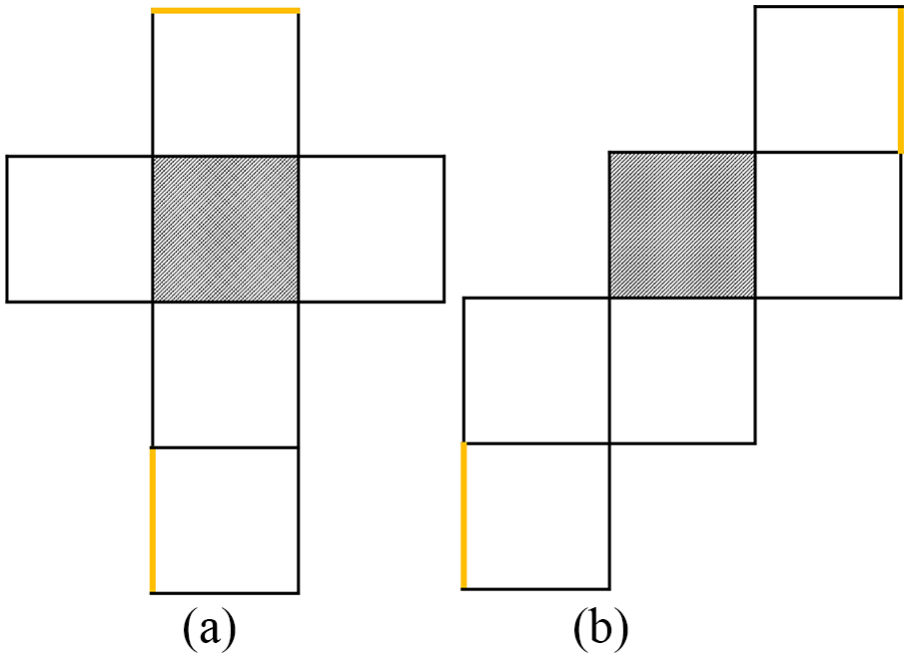
Illustration of the stimuli in the Mental Paper Folding Task. (a) The grid requires three folds. The highlighted (thicker yellow) lines do not overlap in the cube. (b) The grid requires five folds. The highlighted (thicker yellow) lines overlap in the cube.

Each participant performed three versions of the Mental Paper Folding Task which were preceded each by four familiarisation trials with easier stimuli (Dahm & Draxler, 2022) that were not included in the main assessment. In the Mental Paper Folding Task (Dahm & Draxler, 2022), participants were shown a grid of six squares (see Figure 1). In the *Virtual Imagery* version, the grid was shown on the computer screen. Participants were told that the grid represents a piece of paper that can be folded into a cube. Two lines in the grid were highlighted, and participants were asked to evaluate whether these lines would overlap in the cube. Responses were given by using the “left arrow” key (overlap) or the “right arrow” key (no overlap). To preserve the implicitness of the task, imagery instructions or strategies to solve the task were not provided. Further, participants received no instructions regarding hand or head movements. However, participants were required to respond as fast and correctly as possible.

**Figure 2.**
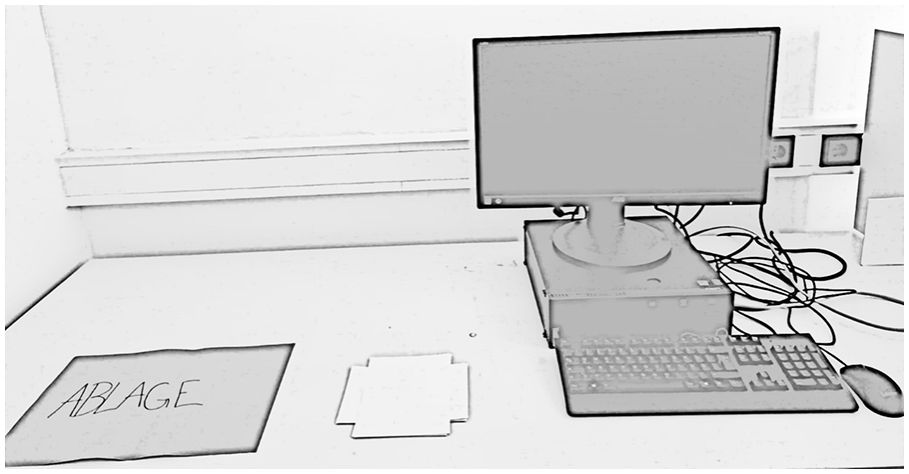
Depiction of the setting in the Realistic Imagery version of the Paper Folding Task with the covered stimulus material besides the keyboard.

In the *Realistic Imagery* version of the Mental Paper Folding Task, a grid of squares was presented on real paper (Figure 2). To assess RTs, participants were instructed to press the “down arrow” key (with the right hand) when they withdrew a paper cover that was placed above the paper stimulus (with the left hand). An experimenter was present throughout data collection to ensure participants were accurate in withdrawing the paper and the simultaneous key-pressing. Participants were instructed not to touch the paper with the grid. All other instructions were equivalent to the Virtual Imagery version. To avoid possible order strategies, the order of the items was reversed in the Realistic Imagery version.

**Figure 3.**
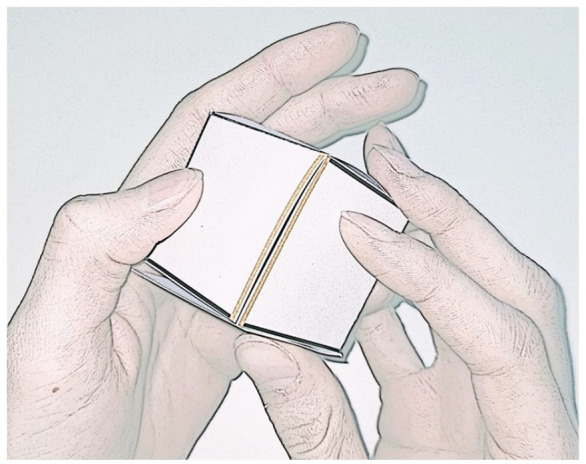
Depiction of the executed version of the Paper Folding Task with the highlighted (yellow) edges overlapping in the cube.

In the *Execution* version of the Mental Paper Folding Task, the folding of the paper was actually *executed* (Figure 3). To assess RTs, participants were instructed to press the “down arrow” key when they withdrew a paper cover that was placed above the paper stimulus. Participants were instructed to hold the stimulus paper in their hands and to fold the paper until one of the highlighted lines overlapped with another edge (see Figure 3). Again, they responded with the arrow keys to indicate overlap or no overlap. In both realistic versions, the paper was pre-folded, to ease up the (imagined) folding action and avoid unnecessary (imagined) creasing actions. The pre-folds were at the same position as the black lines (see Figure 1). The thickness of the paper was 160 g/m². We aimed to investigate the involvement of action imagery in Mental Paper Folding which is usually not executed and used without additional materials (real paper). Because the order of action execution and action imagery may have an impact on durations (Campos et al., 2009; Courtine et al., 2004), the imagery versions of the task were placed before the execution version in the present study. Further, to avoid familiarisation with the realistic material in the virtual version, the Virtual Imagery version was always presented first.

After the Realistic Imagery version and after Execution, participants rated single items based on the Sport Imagery Ability Measure (Ruiz et al., 2019; Watt et al., 2004) on a scale from 1 (not at all) to 9 (very much) whether they (a) perceived (kinesthetic) feelings of their hands, (b) perceived folding actions of their hands visually, (c) perceived a folding of the paper visually without their hands, (d) perceived acoustic aspects of the folding, and (e) were concentrated. Further, they rated (f) the ease and (g) the vividness of (mental) paper folding from 1 (very difficult/no imagery) to 9 (very easy/as vivid as in reality). The items for Ease and Vividness did not differentiate between modalities. To avoid confounding the imagery focus of the Realistic Imagery version, these single items were not presented after the Virtual Imagery version.

Finally, intelligence was assessed using the six-item short version of the Hagen Matrices Test (Heydasch et al., 2013, 2020). To ensure high data quality (see participants section), inattention was assessed at the end of the experiment using the short versions of the infrequency and inconsistency scales (Maniaci & Rogge, 2014).

### Data analysis

Statistical significance was set at *p* < .05. RT was defined as the interval between the availability of the stimulus grid (down arrow key in realistic versions) and participants’ overlapping decisions (left or right arrow keys). The ER indicates the percentage of incorrect responses. Median RTs and the percentage of errors were calculated for each number of Folds (3, 4, or 5) and Task version (Virtual Imagery, Realistic Imagery, Execution). Multivariate Shapiro–Wilk tests (Kassambara, 2021) revealed a significant difference from normal distribution for all ER (*W* ⩽ 0.55, *p* ⩽ .001), but not for most RTs (*W* ⩾ 0.95, *p* ⩾ .186, except for Virtual Imagery with 3 and 4 Folds: *W* ⩽ 0.92, *p* ⩽ .036). The ERs revealed generally little variance and floor effects, with many participants showing less than two errors distributed in violin plots in the online Supplementary Material A.

An ANOVA with the within-factors *Task version* (Virtual Imagery, Realistic Imagery, Execution) and *Folds* (3, 4, 5) was calculated for RTs. For the ANOVA, partial eta squared (η_p_^2^) is reported as effect size (Kassambara, 2021). If Mauchly’s test indicated that the assumption of sphericity was violated, we report Greenhouse-Geisser corrected degrees of freedom and *p*-values. Further comparisons were conducted using *t*-tests with Holm-adjusted pairwise comparisons with Cohen’s *d* as effect size.

Further, to correlate performance in the Paper Folding Task with intelligence (Heydasch et al., 2020), we calculated Linear-Speed Accuracy (LISA) scores that combine RT and ERs (Vandierendonck, 2017, 2021) Because RTs are usually skewed and have outliers (Ulrich & Miller, 1994), we used the median and median absolute deviation (MAD; instead of the mean and standard deviation) when calculating these scores. Hence, either the median RT of correct responses (if the ER is 0%) or median RT of correct responses + ER × MAD (RT)/*SD* (ER) was used. Moreover, Pearson correlations were calculated between task versions (Virtual Imagery, Realistic Imagery, and Execution). In addition, Spearman correlations within each task version between RT, ER, and linear-speed accuracy scores (LISAS) are shown in the online Supplementary Material B.

## Results

### Response Times

**Figure 4.**
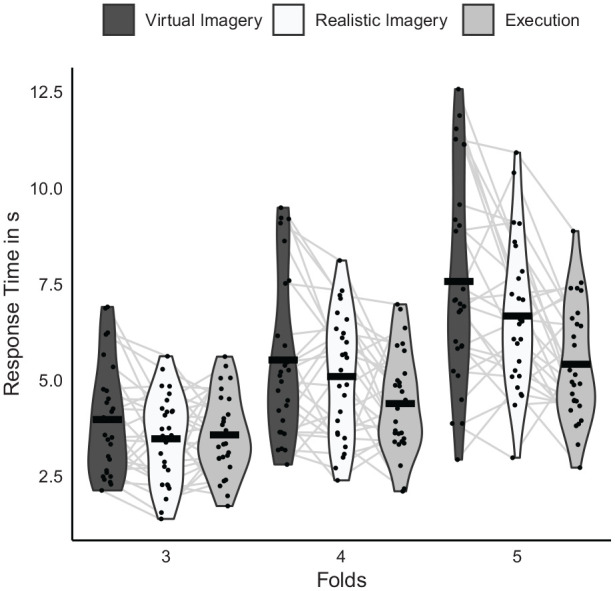
Violin plots and means of response times (RTs) separately for task version (virtual imagery, realistic imagery, and execution) and the number of folds (3, 4, 5).

Violin plots and means of RTs are shown in Figure 4. The significant main effect of *Folds*, *F*(1.3, 34.7) = 166.9, *p* < .001, η_p_² = .87, indicated significantly longer RTs with five folds (*M* = 6.5 s) than with four folds (*M* = 5 s, *p* < .001, *d* = 2.21), and significantly shortest with three folds (*M* = 3.7 s, *p* ⩽ .001, *d* ⩾ 2.15). The significant interaction between *Folds* and *Task version*, *F*(4, 104) = 8.6, *p* < .001, η_p_² = .25, indicated that these differences between *Folds* were significantly more pronounced in the imagery versions than in the execution version (*p* ⩽ .001, *d* ⩾ 0.5), while the differences in the imagery versions did not significantly differ from each other (*p* = .37, *d* = 0.12).

The significant main effect of *Task version*, *F*(2, 52) = 9.3, *p* < .001, η_p_² = .26, was modified by the significant interaction between *Folds* and *Task version*, *F*(4, 104) = 8.6, *p* < .001, η_p_² = .25. This indicated that with three folds RTs did not significantly differ between task versions (*p* ⩾ .138, *d* ⩽ 0.4; Virtual Imagery: *M* = 4 s, Realistic Imagery: *M* = 3.5 s, Execution: *M* = 3.6 s). With four folds, RTs in the Virtual Imagery version (*M* = 5.5 s) were significantly longer than in the Execution version (*M* = 4.4 s, *p* = .004, *d* = 0.6), while RTs in the Realistic Imagery version did not significantly differ from the other two versions (*M* = 5.1 s, *p* ⩾ .038, *d* ⩽ 0.42). Further, with five folds RTs were significantly longer in the Virtual Imagery version (*M* = 7.5 s) than in the Realistic Imagery version (*M* = 6.7 s, *p* = .03, *d* = 0.44) and significantly shorter in the Execution version (*M* = 5.4 s, *p* ⩽ .001, *d* ⩾ 0.69).

### Modality and perspective ratings

**Figure 5.**
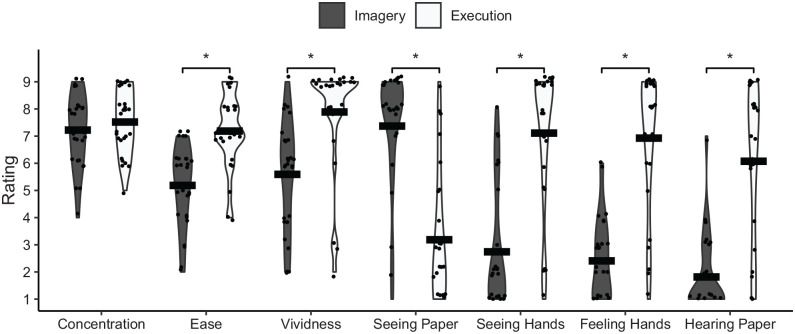
Violin plots and means of the ratings for task-specific single items after performing Realistic Imagery or Execution.

Holm-corrected t-tests were calculated to compare the ratings of the single items (strength of visual and kinesthetic representations, Vividness, Ease, and Concentration) after Realistic Imagery and Execution. Violin plots of the ratings are shown in Figure 5.

Concentration did not significantly differ between Realistic Imagery and Execution, *t*(27) = 1.2, *p* = .256, *d* = 0.22. Execution was rated significantly easier, *t*(27) = 5.1, *p* < .001, *d* = 0.97, and more vivid, *t*(27) = 4.9, *p* < .001, *d* = 0.94, than Realistic Imagery. Similarly, the ratings were significantly stronger in Execution than in Realistic Imagery for visual imagery from a first-person perspective through one’s own eyes (seeing the own hands), *t*(27) = 7.5, *p* < .001, *d* = 1.44, kinesthetic imagery (feeling the hands), *t*(27) = 7.3, *p* < .001, *d* = 1.4, and acoustic imagery (hearing the paper), *t*(27) = 7.1, *p* < .001, *d* = 1.36. In contrast, visual imagery from a third-person perspective without the hands of an actor (seeing the paper being folded), *t*(27) = −6.3, *p* < .001, *d* = −1.2, was rated significantly stronger in Realistic Imagery than in Execution.

### Correlations

Correlations between intelligence (Heydasch et al., 2020) and the LISA scores in Mental Paper Folding showed a tendency of higher intelligence scores with better Mental Paper Folding Performance in imagery (Virtual Imagery: *r* = −.2; Realistic Imagery: *r* = −.55; Execution: *r* = −.08). To investigate Mental Chronometry in the Paper Folding Task, we calculated Pearson correlations between Virtual Imagery, Realistic Imagery, and Execution (Table 1). This revealed significant correlations between imagery and execution in RTs and LISAS but not in ER.

**Table 1.** Correlations between the task versions (virtual imagery, realistic imagery, and execution) in response times (RT), error rates (ER), and linear-speed accuracy scores (LISAS).

Correlation	RT	ER	LISAS
	Pearson	Spearman	Pearson
Virtual Imagery × Realistic Imagery	**.67**	.37	**.55**
Virtual Imagery × Execution	**.5**	.02	**.47**
Realistic Imagery × Execution	**.43**	−.08	**.42**

*Note*. with *N* = 28 is *r*_crit_ = .367 and ρ_crit_ = .375. Significant correlations are shown in bold.

Further, we analysed the correlations of reported subjective and objective measures (Table 2), which revealed a significant correlation between the rated Ease of the task and LISA scores in Realistic Imagery, a significant correlation between Seeing their Hands and LISA scores in Realistic Imagery, and a significant correlation between Seeing the Paper and LISA scores in Execution.

**Table 2.** Pearson correlations between reported subjective measures and Linear-Speed Accuracy Scores (LISAS) in realistic imagery and execution.

Rating	Imagery LISAS	Execution LISAS
Concentration	−**.46**	−**.37**
Ease	−**.42**	.13
Vividness	.34	.1
Seeing Paper	−.28	**.4**
Seeing Hands	**.48**	.2
Feeling Hands	.24	.14
Hearing Paper	−.18	.14
External Visual Imagery	.05	.19
Internal Visual Imagery	−.03	−.09
Kinesthetic Imagery	−.12	.03

*Note*. With *N* = 28 is *r*_crit_ = .367.

Significant correlations are shown in bold.

## Discussion

Using mental chronometry, we examined the use of imagery in a Paper Folding Task by comparing imagery durations with execution durations. Although stronger in imagery than in execution, the number of folds constrained performance in the Paper Folding Task in both, imagery and execution. Hence, the same principle constrained both imagery and execution, but not to the same degree. Further, performance in the Paper Folding Task correlated between imagery and execution, strengthening the assumption of similar mechanisms and the involvement of action imagery. Along with the expectations, correlations of imagery ability scores were observed with intelligence and some subjective single items.

### Mental chronometry

The comparison of task versions showed that RTs were longer in Virtual Imagery and Realistic Imagery than in Execution. Longer durations in action imagery than in action execution go along with several other tasks such as bimanual reaching (Dahm & Rieger, 2016a, 2016b), visually guided pointing (Cerritelli et al., 2000), golf putting (Orliaguet & Coello, 1998), and short walking distances (Grealy & Shearer, 2008). It has been argued that action imagery durations are longer than action execution durations in complex actions (Guillot & Collet, 2005) and especially in short (< 3 s) actions (Grealy & Shearer, 2008; Guillot & Collet, 2005). Further, it is assumed that action imagery durations are prolonged due to the fact that action processes that are implicit and automatised in execution need to be made consciously available in action imagery (Glover & Baran, 2017).

Besides differences in absolute timing, the impact of constraints on action imagery durations and action execution durations is often similar (Dahm & Rieger, 2016a, 2016b; Guillot, Hoyek, et al., 2012). However, in the present study, the number of folds had a stronger impact on action imagery durations than on action execution durations. This stands in contrast to the functional similarity assumption (Jeannerod, 2001). Similar findings have been reported for walking with a 25 kg load (Decety et al., 1989; Munzert et al., 2015), which increased action imagery durations but not action execution durations. While the load conditions required additional effort that compensated for an increase in action execution durations (Decety et al., 1989), compensation effects are not plausible in the present study. Still, the impact of the folding constraint was overestimated in action imagery compared to action execution. This can be explained by the motor-cognitive model of action imagery (Glover & Baran, 2017), which states that action imagery is decelerated when executive functions are engaged (high cognitive load) more strongly in action imagery than in action execution. A possible reason why task difficulty had a lower impact on execution is that after folding the paper (in execution), the overlapping pieces are simply overt. In contrast, during (action) imagery, it is necessary to track the previous folds to follow up with the next folds (working memory load) which most likely impacts RTs (Glover et al., 2020). What further strengthens this argument is that the finding of longer RTs in imagery than in execution was observed with five folds but not with three folds. Five folds increase the working memory load more than three folds as more steps need to be kept up with for a longer time. This highlights the importance of the Imagery Maintenance dimension (Cumming & Eaves, 2018) in Mental Paper Folding.

What stands in favour of functional similarity (Glover & Baran, 2017; Jeannerod, 2001) are the strong correlations between action imagery durations and action execution durations. Such correlations between action imagery and action execution have previously been observed in bimanual reaching durations (Dahm & Rieger, 2016b) and dart-throwing accuracy (Dahm & Rieger, 2019). Hence, the correlations make us assume that action imagery and action execution are based on similar mechanisms such as internal models (Rieger et al., 2023).

### Objective measures and subjective ratings

The comparisons between Mental Paper Folding Performance and subjective imagery ratings revealed a significant correlation between the rated ease of mentally folding the paper and the Mental Paper Folding Performance. Hence, participants were able to judge whether it was difficult for them to solve the task or not. However, in contrast to our expectations, participants’ ratings of their general action imagery abilities from the Vividness of Movement Imagery Questionnaire (Dahm, 2022; Roberts et al., 2008) did not correlate with Mental Paper Folding Performance. If both measures assess the same construct (action imagery ability), medium to strong correlations would have been expected. One explanation could be that self-ratings are biased in questionnaires due to social desirability and self-protection (Gabbard & Lee, 2014) or because it is not possible to classify your own ability if you do not know others’ abilities. An alternative explanation could be that the measures involve different subdimensions of action imagery ability (ease vs. vividness). However, small correlations would still have been expected as they are both subsumed under general action imagery ability (Dahm, 2020). For instance, ratings for the ease of action imagery in the Movement Imagery Questionnaire (Williams et al., 2012) correlate with ratings of the vividness of action imagery in the Vividness of Movement Imagery Questionnaire (Dahm, 2022; Roberts et al., 2008). A third alternative is that Mental Paper Folding may not measure action imagery ability. But what does it measure instead? The single items revealed that in the imagery version of the Paper Folding Task (without execution), individuals focus more strongly on seeing the paper being folded than focusing on their own hands folding the paper. Hence, they may rely on *visual imagery of a dynamic object* rather than imagining their own movements. The correlations between the single items and the LISA scores further strengthen this argument, indicating that focusing on the hands is detrimental to performance in imagery. In the same way, focusing solely on paper folds without a focus on the hands is detrimental to performance in executed paper folds.

### Limitations and future directions

The ratings in the single items were assessed only after the Realistic Imagery version but not after the Virtual Imagery version. Therefore, we cannot rule out the possibility that Virtual Imagery ratings might have differed. However, it seems unlikely that the Virtual Imagery version provokes stronger feelings in the own hands than the Realistic Imagery version. The ratings revealed that during action execution participants perceived themselves as actors which helped them to have a vivid first-person perception of seeing their hands, feeling their hands, and hearing the paperfolds. In contrast, during action imagery, participants perceived no actor, but rather the paper folding itself. In the present study, we aimed to investigate perspective and actor in implicit Mental Paper Folding. Therefore, no explicit instructions to perform action imagery were given. It remains an open question, whether instructions that focus on the feeling of imagining oneself while folding the paper would reinforce a first-person perspective in the Mental Paper Folding Task which might result in stronger correlations with existing action imagery questionnaires. It would be interesting to test this assumption in future studies in relation to other visual measures that involve a dynamic imagery component without motor components (Mental Object Rotation) or with motor components (Mental Body Rotations) (Parsons, 1987; Shepard & Metzler, 1971; Steggemann et al., 2011; Vingerhoets et al., 2002) because even object rotations may involve motor processes (Wexler et al., 1998; Wohlschläger & Wohlschläger, 1998).

We had expected correlations of action imagery ability with intelligence because both constructs overlap in the nomological net (Dahm, 2020; Tong, 2013). However, this was only partially observed. The intelligence task (Heydasch et al., 2020) which involved visual-spatial intelligence, revealed correlations with the Paper Folding Task only in the Realistic Imagery version. Future studies could use other measures of visual-spatial working memory (e.g. Corsi blocks, visual-spatial n-back task) that, according to the Motor-Cognitive Model (Glover et al., 2020), should be correlated with Mental Paper Folding.

It has been claimed that action expertise may influence action imagery quality (Campos et al., 2009; Courtine et al., 2004). In the present study, we cannot rule out the possibility that participants had previous experience in paper folding (e.g. origami folding). However, we assume that expertise was low as folding a cube from a two-dimensional sheet of paper is not a common task in daily life. Further, to reduce experience effects, the order of the conditions was kept constant for all participants. Still, future studies may investigate the effects of execution knowledge on Mental Paper Folding Performance.

## Conclusion

The Mental Chronometry Tests indicated that the number of folds increased RTs in both imagery and execution. However, this increase was stronger in imagery than in execution, indicating an overestimation of the folding constraint in action imagery. While the positive correlations between imagery durations and execution durations lend support to the assumption of action imagery processes in the Mental Paper Folding Task, the single items indicated that Mental Paper Folding involves *visual imagery of dynamic objects* rather than imagery of one’s own action. Further, performance in the Mental Paper Folding Task did not correlate with subjective action imagery ability ratings. Therefore, the Mental Paper Folding Task may be considered as an (action) imagery ability measure different from the existing questionnaires, particularly for assessing visual dynamic imagery of actions without an actor or imagery of action consequences.

## Supplemental Material

sj-pdf-1-qjp-10.1177_17470218241249727 – Supplemental material for Let’s do it: Response times in Mental Paper Folding and its execution Supplemental material, sj-pdf-1-qjp-10.1177_17470218241249727 for Let’s do it: Response times in Mental Paper Folding and its execution by Stephan Frederic Dahm and Pierre Sachse in Quarterly Journal of Experimental Psychology
