# Supplementary material for: Let’s do it: Response times in Mental Paper Folding and its execution
Source: Q J Exp Psychol (Hove). 2024 May 12;78(4):731–43. doi: 10.1177/17470218241249727 (PMC11905326; doi:10.1177/17470218241249727)
Supplement: sj-pdf-1-qjp-10.1177_17470218241249727 – Supplemental material for Let’s do it: Response times in Mental Paper Folding and its execution [file sj-pdf-1-qjp-10.1177_17470218241249727.pdf]

Supplementary Material for:

Let’s do it: Response times in Mental Paper Folding and its execution.

Stephan Frederic Dahm & Pierre Sachse

University of Innsbruck, Faculty of Psychology and Sports Sciences, Department of Psychology

Corresponding author
Stephan F. Dahm
University of Innsbruck, Department of Psychology
Universitätsstraße 5-7, 6020 Innsbruck, Austria
stephan.dahm@uibk.ac.at

Supplementary Material A

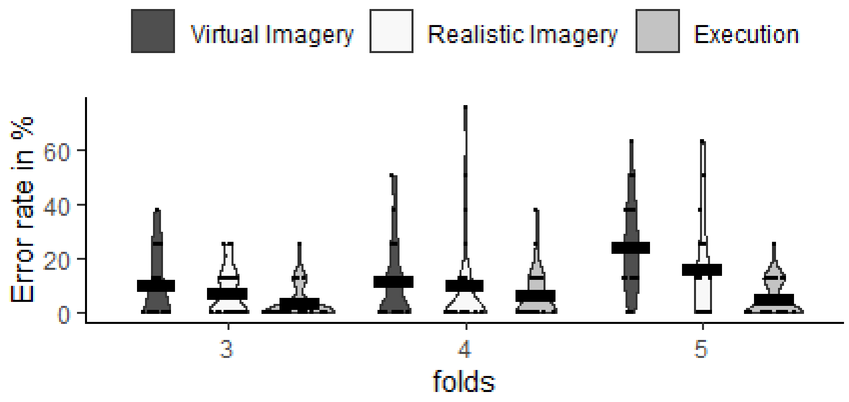

Figure SM1.
Violin plots of Error rates separately for Task version (Virtual Imagery, Realistic Imagery, and Execution) and the Number of Folds (3, 4, and 5).

Supplementary Material B

To assess the relative impact of RTs and ERs on the combined LISA score, we calculated Spearman correlations between RT, ERs, and LISAS within each task version. A Fishers z test revealed that the LISA score correlated significantly stronger with RTs ($r \geq .87$) than with ERs ($r \leq .33$, $z = 3.5$, $p < .001$).

These correlations aimed to investigate the interpretation of the LISA scores (Vandierendonck, 2021) that were used in the present study in Mental Paper Folding. The LISA scores were heavily correlated with RTs and only little with ER. One reason could be that the ER revealed generally less variance and floor effects (with many participants showing less than 2 errors). Hence, LISA scores in Mental Paper Folding can be regarded as a measure of mainly RTs. However, in the LISA score participants are penalized for making errors favoring those subjects who perform correctly.

Spearman correlations between Response Times (RT), Error rates (ER), and Linear-Speed Accuracy Scores (LISAS).

Correlation	Virtual Imagery	Realistic Imagery	Execution
RT x LISAS	**.88**	**.87**	**.97**
ER x LISAS	.11	.33	.25
RT x ER	.13	.14	.22

Note. with *N* = 28 is ρ_{crit} = .375. Significant correlations are shown in bold.
